# Presence of activating *KRAS *mutations correlates significantly with expression of tumour suppressor genes *DCN *and *TPM1 *in colorectal cancer

**DOI:** 10.1186/1471-2407-9-282

**Published:** 2009-08-13

**Authors:** Vid Mlakar, Gašper Berginc, Metka Volavšek, Zdravko Štor, Miran Rems, Damjan Glavač

**Affiliations:** 1 Department of Molecular Genetics, Institute of Pathology, Faculty of Medicine, University of Ljubljana, Ljubljana, Slovenia; 2 Department of Abdominal Surgery, University Medical Centre Ljubljana, Ljubljana, Slovenia; 3 Department of Surgery, Jesenice Hospital, Jesenice, Slovenia

## Abstract

**Background:**

Despite identification of the major genes and pathways involved in the development of colorectal cancer (CRC), it has become obvious that several steps in these pathways might be bypassed by other as yet unknown genetic events that lead towards CRC. Therefore we wanted to improve our understanding of the genetic mechanisms of CRC development.

**Methods:**

We used microarrays to identify novel genes involved in the development of CRC. Real time PCR was used for mRNA expression as well as to search for chromosomal abnormalities within candidate genes. The correlation between the expression obtained by real time PCR and the presence of the *KRAS *mutation was investigated.

**Results:**

We detected significant previously undescribed underexpression in CRC for genes *SLC26A3*, *TPM1 *and *DCN*, with a suggested tumour suppressor role. We also describe the correlation between *TPM1 *and *DCN *expression and the presence of *KRAS *mutations in CRC. When searching for chromosomal abnormalities, we found deletion of the *TPM1 *gene in one case of CRC, but no deletions of *DCN *and *SLC26A3 *were found.

**Conclusion:**

Our study provides further evidence of decreased mRNA expression of three important tumour suppressor genes in cases of CRC, thus implicating them in the development of this type of cancer. Moreover, we found underexpression of the *TPM1 *gene in a case of CRCs without *KRAS *mutations, showing that *TPM1 *might serve as an alternative path of development of CRC. This downregulation could in some cases be mediated by deletion of the *TPM1 *gene. On the other hand, the correlation of *DCN *underexpression with the presence of *KRAS *mutations suggests that *DCN *expression is affected by the presence of activating *KRAS *mutations, lowering the amount of the important tumour suppressor protein decorin.

## Background

Colorectal cancer (CRC) is the third most common cancer in both men and women [1]. It remains one of the most frequent and deadly diseases [1] despite important advantages in treatment and diagnosis [2]. The evolution from normal colonic mucosa to adenoma with different grades of dysplasia and finally to invasive cancer is associated with a series of genetic events occurring over a long period [3]. Not surprisingly, the incidence of colorectal cancer increases with age, especially after 60 years. Two pathways have been proposed for colorectal cancer development. The microsatellite instability (MSI) pathway is caused by mutations in genes *MLH1*, *MSH2*, *PMS2 *and *MSH6*, which are part of the mismatch repair system. The chromosomal instability (CIN) pathway is associated with mutations in the APC gene or loss of 5q (APC) and mutations in the *KRAS *gene during progression of normal epithelium to early adenoma [4]. Loss of 18q (*SMAD2*, *SMAD4*, *DCC*) is associated with progression from early to advanced adenoma [4]. Deletion of 17p, which contains the *p53* gene, is involved in progression towards cancer [4]. However, only a minority of CRCs possess a full spectrum of these molecular abnormalities [5]. Moreover, even though major genes involved in the development of colorectal cancer have been identified, interestingly most of them have shown only limited value in clinical use [6]. Detection of germline mutations in *MLH1*, *MSH2*, *PMS2*, *MSH6 *and APC is used to identify individuals with a predisposition for developing hereditary nonpolyposis colon cancer (HNPCC) and familial adenomatous polyposis (FAP), respectively. However, only a small percentage of the population are carriers of these mutations [7,8]. On the other hand, somatic mutations in *p53*, *APC *and *KRAS *have no real prognostic value in cases of sporadic colorectal cancer [6]. Nevertheless, some clinical use might come from mutations in *KRAS*. They have recently been shown to be involved in the response of CRCs to cetuximab, suggesting a better response of patients without *KRAS *mutations in CRCs [9,10]. This shows that several steps in CRC development might be bypassed by other as yet unknown genetic events that lead towards CRC. We therefore wanted to improve our understanding of the genetic mechanisms of CRC development. In turn, this might identify new potential tumour markers useful for clinical practice.

## Methods

### Samples

Sixteen samples of colorectal adenocarcinoma (CRC) and corresponding normal tissue were collected at the Department for Surgery at Jesenice hospital. Samples were stabilized in RNAlater (Ambion, USA) solution immediately after extraction. Samples were incubated at 4°C overnight and stored at -20°C, according to the manufacturer's recommendations. The study was approved by the National Medical Ethics Committee of the Republic of Slovenia. All 16 samples were evaluated by an expert pathologist at the Institute of Pathology, Faculty of Medicine, University of Ljubljana. Adjacent to samples stored in RNAlater tissue sections were obtained for histological evaluation. Only samples including within the range of 60% to 90% of invasive tumour cells were accepted for further analysis. Corresponding normal tissue samples contained no cancer cells as evaluated by pathologist. Tumour stage and nodal status were determined for each patient (Table 1).

**Table 1:** Samples, histology and presence of activating *KRAS *mutations

Number	T	N	*KRAS *status
1	3	0	wt

2	1	0	wt

3	2	0	wt

4	4	2	wt

5	3	2	wt

6	3	1	12G>S

7	3	2	13G>D

8	1	0	12G>S

9	3	1	13G>D

10	4	0	13G>D

11	3	1	wt

12	3	1	wt

13	3	0	12G>S

14	3	0	12G>S

15	3	0	13G>D

16	3	0	wt

The table shows sample histological data and the presence of activating *KRAS *mutations. No activating KRAS mutations were found in corresponding normal tissue.

T = tumour stage, N = nodal status, *KRAS *status – presence or absence of activating *KRAS *mutations.

### DNA extraction

DNA from tissue stabilized in RNAlater (Ambion, USA) was isolated with a QIAgen kit (QIAgen) according to the manufacturer's instructions.

### RNA extraction

RNA from tissue was isolated using TRI REAGENT™ (Sigma Aldrich) and the PureLinkTM Micro-to-Midi Total RNA Purification System (Invitrogen), according to the manufacturer's instructions. Briefly, 50 – 100 mg of tissue was homogenized in TRI REAGENT™ using PT 3100 homogenizer (Polytron). The homogenate was centrifuged at 12000 × g for 10 min at 4°C to remove insoluble material and the supernatant was transferred to a fresh microcentrifuge tube. Samples were allowed to sit at room temperature for 5 min, and 0.2 ml of chloroform was added per 1 ml of TRI REAGENT™. Samples were mixed by hand for 15 s and allowed to stand for 2 – 15 min at room temperature. The resulting mixture was centrifuged at 12000 × g for 15 min at 4°C. The aqueous phase was transferred to a fresh microcentrifuge tube and an equal amount of 70% ethanol was added. Samples were transferred to a PureLinkTM Micro-to-Midi Total RNA Purification System column (Invitrogen) and processed according to the manufacturer's protocol. All samples were washed from the column with 75 μl of RNAse free water.

### Analysis of RNA

The quality of RNA was checked on a Bioanalyzer 2100 (Agilent) using an RNA 6000 Nano Labchip (Agilent) and a 6000 RNA ladder as reference (Ambion). The concentration and quantity of RNA was determined with ND-1000 (Nanodrop, USA).

### Preparation of aaRNA

Preparation of aaRNA was performed with an Amino Allyl MessageAmp™ II aRNA Amplification Kit (Ambion) according to the manufacturer's recommendations. For each hybridization, we labelled 5 μg of normal (Cy3) and 5 μg of tumour (Cy5) mRNA. After removing the excess dye, the RNAs were dissolved in Nexterion Hybridization solution (Schott Nexterion).

### Microarrays

Microarrays were prepared with Human Apoptosis Subset v2.0 and Human Cancer Subset v3.0 (Operon) 70mer oligonucleotides and Nexterion 70mer Oligo Microarraying Kit (Schott Nexterion) slides. Oligonucleotides were spotted using an MG1000 spotter (MicroGrid), immobilised and stored according to the manufacturer's instructions (Schott Nexterion). All hybridisations were performed on HS400 (Tecan) according to the manufacturer's instructions (Schott Nexterion). We used an LS200 scanner (Tecan) at 6 μm resolution for scanning the microarrays.

### Real time PCR

We used Taqman Reverse transcription reagent (Applied Biosystems) for cDNA synthesis. Expression of the *SLC26A3*, *DCN*, *CALM3 *and *TPM1 *genes in tumour samples relative to their normal adjacent tissues was measured using quantitative real time PCR based on the TaqMan^® ^fluorescence methodology. A ready mixture of probes and primers specific for *SLC26A3 *mRNA expression (Assay-on-Demand™, Hs00995363_m1 (Applied Biosystems)), *DCN *mRNA expression (Assay-on-Demand™, Hs00266491_m1 (Applied Biosystems)), *CALM3 *mRNA expression (Assay-on-Demand™, Hs00270914_m1 (Applied Biosystems)), *TPM1 *mRNA expression (Assay-on-Demand™, Hs00165966_m1 (Applied Biosystems)) and Pre-Developed TaqMan Assay Reagents Human GAPDH (20×) mRNA (Applied Biosystems) were used as the endogenous control gene. All measurements were done in triplicate. The relative quantification of mRNA levels of the target was determined using the ΔΔC_T _(ΔΔC_T _= ΔC_T normal_-ΔC_T tumour_; ΔC_T _= ΔC_Tx_-ΔC_T GAPDH_) method, unless stated otherwise.

### Copy number

Gene copy number was determined by using real time PCR based on the TaqMan fluorescence methodology. The assays, TaqMan RNase P Detection Reagents (Applied Biosystems) and Assay-by-Design for *TPM1*, *SLC26A3 *and *DCN *(Applied Biosystems) gene detection were performed according to the manufacturer's instructions. Assay-by-Design for the *TPM1 *gene contained the following primers and probe: forward primer: GGAAAGTACATATCTGGGAGAAGCA, reverse primer: TTCTTGATGGCGTCCATGGT, probe: FAM TCGCACTCCCGCTCCT. Assay-by-Design for the *SLC26A3 *gene contained the following primers and probe: forward primer: GCCACAGCCAACAGAAAAATCAAAAT, reverse primer: CCTCAAAAGCATTTGTAGAATACACTGG, probe: FAM CTGGCCACAATATAC. Assay-by-Design for the *DCN *gene contained the following primers and probe: forward primer: CTGATGACCGCGACTTCGA, reverse primer: CGAAGATGGCATTGACAGCG, probe: FAM CCCAGTGTGCCCCTTC. The copy numbers of *TPM1*, *SLC26A3 *and *DCN *genes were determined on the basis of the threshold cycle difference between *TPM1*, *SLC26A3 *and *DCN *and the RnaseP gene. For calculation of the copy number, combined_gene_copy_number_assay_macro-1.xls (Applied Biosystems) was used.

### KRAS mutation detection and V64L (rs17018909) detection

The first exon of the *KRAS *gene of each tumour sample and corresponding normal tissue was amplified with PCR as previously described by Konig and co. [11]. Screening for changes in PCR products was performed with Single Stranded Conformation Analysis (SSCA) [12,13]. Samples showing different migration shifts were chosen for sequencing. All 16 corresponding normal tissues were also sequenced for V64L (C>A, rs17018909) polymorphism of the *DCN *gene, which might have an effect on performance of Hs00266491_m1 Assay-on-Demand™ (Applied Biosystems). Sequencing was performed using BigDye Terminator Ready Reaction Mix. Sequences were purified, dissolved and analysed on an ABI PRISM 310 Genetic Analyzer according to the manufacturer's recommendations (Applied Biosystems, USA). Nucleotide sequences were compared against the published *KRAS *cDNA sequence (GenBank accession number NM_014588) in order to define the mutation.

### Data analysis

We used ArrayPro Analyser (Media Cybernetic, USA) software for feature extraction after imaging. Acuity 4.0 (Molecular Devices, USA) was used for filtration of bad signals, Lowess normalization and microarray data analysis. We filtered out genes that were not expressed in all 16 samples and showed a median expression of 1.5 times. Results on differentially expressed genes were compared to 5 major expression studies available from the PubMed GEO project [14-18] and evidence of independent validation of gene expression data was checked using the PubMed database. All other statistical analyses were done using SPSS 16 (SPSS inc.). We used the paired t test to compare differences in the amount of *TPM1*, *SLC26A3*, *DCN *and *CALM3 *mRNA between CRC and corresponding normal tissue. The paired t test was also used to check the difference in *GAPDH *expression between corresponding normal tissue and tumour samples. The t test was also used to compare pairs of samples with mutations in the *KRAS *gene to pairs of samples without these mutations. P < 0.05 was considered statistically significant.

## Results

The expression profiles of 16 primary human colorectal tumours were obtained by comparison of CRC mRNA with mRNA from their corresponding normal tissue on a 3060 element microarray from Operon. In our experimental design, microarrays were used as a dual colour system in which CRC and the corresponding normal mRNA were separately labelled, mixed and hybridised together on each array. The acquired data were analysed with Acuity 4.0 to select reliable data. Only genes present in all 16 microarrays were considered for further processing. Out of 3060 genes, 30 genes showed differential expression (see additional file 1[14-18]).

Because of the manageable number of differentially expressed genes, we crosschecked their expression to already existing microarray data (see additional file 1). Eleven out of 30 genes (*SLC26A3*, *CASP9*, *VIM*, *LGALS4*, *SRI*, *UGP2*, *LGALS3*, *TPM1*, *TUBB*, *MAP2K7 *and *CRABP2*) showed good concordance with multiple microarray expression studies. Ten out of 30 genes (*ITGB4*, *CALM3*, *EEF1A1*, *HOX4A*, *PCDH1*, *KRT19*, *GATA2*, *TGFBI*, *RBL2*, *and TNA*) showed variable expression between different studies. Interestingly, 3 genes (*AD022*, *CORT*, *and CARD10*) were not included in any of these studies and 4 genes (*DCN*, *NPC1*, *RAI1*, *TTYH1*) were characterised in only 1 study. Only *CDH12 *and *PMP22 *showed a difference between multiple expression studies and our results. In order to enable other users comprehensively to interpret and evaluate our results, original tables of complete microarray results are available in the supplementary data (see the GEO website at http://www.ncbi.nlm.nih.gov/projects/geo/ Series entry: GSE14010).

### Real time PCR

**Figure 1:**
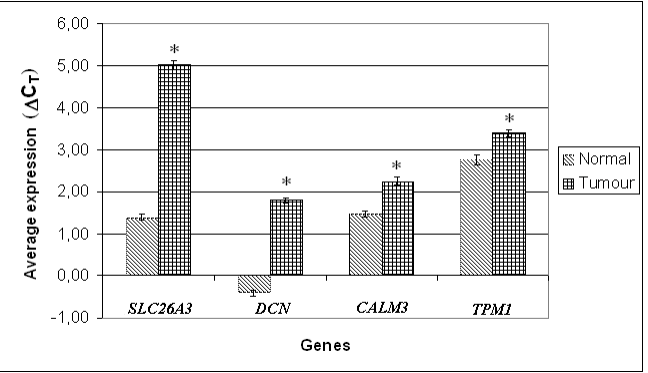
**Significant under-expression of *SLC26A3*, *DCN*, *CALM3 *and *TPM1 *in tumour in comparison to normal tissue**. Average expression of the *SLC26A3*, *DCN*, *CALM3 *and *TPM1 *gene was significantly lower in CRC in comparison to normal tissue. Expression is shown in ΔC_T _units, whereby larger ΔC_T _value represents down-regulation of *SLC26A3*, *DCN*, *CALM3 *and *TPM1 *in comparison to smaller ΔC_T _of normal tissue.

Relative quantification real-time PCR was used to determine the difference in expression of SCL26A3, *DCN*, *TPM1 *and *CALM3 *between CRC and corresponding normal tissue (Figure 1). We used *GAPDH *as endogenous control for normalization. No statistically significant difference in expression of *GAPDH *between corresponding normal tissue and tumour samples was detected (paired t test p = 0,113). These genes were chosen because they have been shown to play a significant role in CRC development. They also showed good concordance with other microarray studies and no precise quantification of their mRNA has yet been performed.

**Figure 2:**
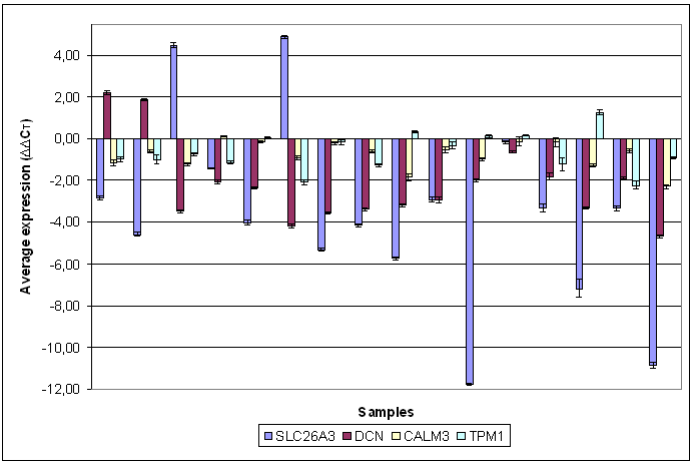
**Expression of *SLC26A3*, *DCN*, *CALM3 *and *TPM1 *in 16 CRC in comparison to normal tissue**. Expression is shown in ΔΔC_T _units, whereby a negative ΔΔC_T _value represents down-regulation of gene in CRC in comparison to corresponding normal tissue. Positive bar represents over-expression of gene in CRC in comparison to corresponding normal tissue.

Figure 1 shows the relative average expression of genes in comparison to normal tissue (Figure 1). In agreement with microarrays, *SLC26A3*, *DCN*, *CALM3 *and *TPM1 *showed clear downregulation. *SLC26A3 *was downregulated in 13 out of 16 cases (Figure 2). Two cases showed overexpression and 1 case showed almost no change in expression. *SLC26A3 *had 12.43 times lower expression in CRC in comparison to normal tissue (p < 0.003). *DCN *was under- and overexpressed in 14 and 2 cases of CRC in comparison to normal tissue, respectively (Figure 2). CRC had on average 4.61 times less *DCN *than normal tissue (p < 0.007). We also report that we did not detect V64L polymorphism in 16 corresponding normal tissues, so no adverse effects on performance of real-time PCR was expected, as suggested by the manufacturer.* CALM3 *showed underexpression in 11 and no change in 5 cases of CRC (Figure 2). CRC had on average 1.7 times less *CALM3 *than normal tissue (p < 0.003). *TPM1 *was underexpressed in 9 out of 16 cases (Figure 2). In only 1 case was *TPM1 *overexpressed in CRC in comparison to normal tissue. The remaining samples showed no major difference. *TPM1 *was on average 1.6 times lower in CRC that normal tissue (p < 0.015). However, when considering only downregulated samples, 2.5 times less *TPM1 *mRNA was found in CRC in comparison to normal tissue.

### Copy number

So far, *SLC26A3*, *DCN *and *TPM1 *genes have been suggested to have tumour suppressor properties. We investigated the possibility that loss of alleles could be present in the location of genes to contribute to underexpression or inactivation of the mentioned genes. We used the real time PCR method to evaluate the amount of allele present in each sample. Interestingly, although we did not find any allele loss at the site of *SLC26A3 *and *DCN *genes, we detected 1 loss of the *TPM1 *allele in 16 samples of CRC (data not shown).

### Mutation detection

**Figure 3:**
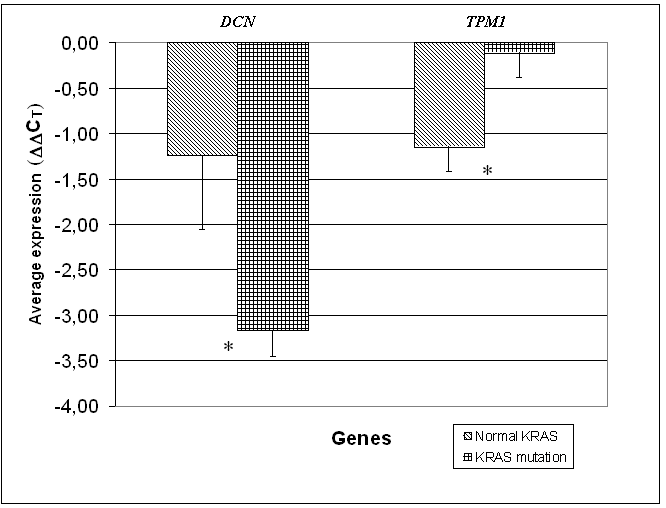
**Expression of *DCN *and *TPM1 *in pairs of samples with a different *KRAS *status**. Expression is shown in ΔΔC_T _units, whereby a negative ΔΔC_T _value represents down-regulation of *DCN*, *TPM1 *in comparison to normal tissue. Pairs of samples were grouped according to their *KRAS *status.

In order to detect mutations, we used the SSCA method for preliminary testing and automatic sequencing for subsequent confirmation of different patterns. We searched for mutations in codon 12 and 13, since these mutations represent 96 – 99% of all mutations detected in CRC. Mutations in other positions, such as 66 and 146, account for around 1–4% of all mutations, although their clinical relevance in CRC is unclear [19]. We found activating mutations in codon 12 and 13 in 8 out of 16 samples (Table 1). No activating *KRAS *mutations were found in corresponding normal tissue. When comparing the expression difference between normal and tumour samples, we found significant underexpression of the *DCN *gene in the group of tumours with activating *KRAS *mutations in comparison with tumours without these mutations (p < 0.05). We also found no change in expression of the *TPM1 *gene in CRC cases with a mutation in *KRAS*, in comparison with underexpression of the *TPM1 *gene in CRCs without activating *KRAS *mutations (p < 0,05) (Figure 3).

## Discussion

Major molecular pathways involved in the development of colorectal cancer have been identified [4]. However, major genes, such as *p53 *and *APC*, involved in these pathways have shown only limited value in clinical use [6]. Moreover, only a minority of CRCs possess a full complement of these molecular abnormalities [5]. This shows that several steps in CRC development might be bypassed by other as yet unknown genetic events that lead towards CRC. Using expression profiling, we set out to identify genes that may play an important role in the development of CRC, whose expression has not yet been characterized. This is also the first study performed on a cohort of Slovenian patients on this scale. We were able to identify 30 differentially expressed genes (see additional file 1). When comparing the obtained results with other studies, only 2 genes showed a large difference in expression in comparison to our study. It is noteworthy that 10 out of 30 genes showed variable expression between studies. Even more interesting was the fact that we were able to find better characterisation of expression with other methods than microarrays for only 7 of 30 genes for CRC samples (see additional file 1). These two observations show the need to characterise expression further with other, more precise methods, such as real time PCR.

In the group of genes showing good concordance with other microarray studies were *SLC26A3*, *CASP9*, *VIM*, *LGALS4*, *SRI*, *UGP2*, *LGALS3*, *TPM1*, *TUBB*, *MAP2K7*, and *CRABP2*. *DCN *and *RAI1 *also showed concordance with our results, although their expression was characterised in only one study. When we looked for the function of these genes, we found that three (*SLC26A3*, *TPM1*, and *DCN*) of them play a tumour suppressor role. Moreover, no precise expression analysis on clinical cases of CRC exists to confirm their involvement. *TPM1 *and *DCN *are also related to the *KRAS *signalling pathway, suggesting that their expression might change due to the presence or absence of activating *KRAS *mutations in codon 12 and 13. Moreover, the expression of none of the three genes has so far been characterised with real time PCR in samples of CRC.

The *SLC26A3 *gene encodes anion transporter expressed in mucosa of the lower gastrointestinal tract [20]. Mutations in the *SLC26A3 *gene cause congenital chloride diarrhea (CLD) [21]. This rare condition is attributed to a lack of chlorine uptake in the colon [22]. Patients with CLD also have a higher risk of developing CRC [23]. Chapman et al also demonstrated that *SLC26A3 *possesses a tumour suppressor ability by inhibiting cell growth in cells transfected with functional *SLC26A3*. Moreover, they showed that the tumour suppressor role is separated from the role of anion transporter [24]. This suggests that *SLC26A3 *is not downregulated as a result of transition of normal colon mucosa towards CRC, but plays an active role in CRC development. The few studies investigating expression of *SLC26A3 *so far have only used subtractive hybridization and northern blot to show that *SLC26A3 *is downregulated in adenomas of colon. Using northern blot analysis, Anthalis et al. observed progressive downregulation of *SLC26A3 *from normal mucosa to polyp and finally to CRC [25]. These methods are not as precise as the real time PCR method used in this study, which provides more accurate and reproducible quantification with a larger dynamic range. In agreement with above mentioned data, we report underexpression of *SLC26A3 *in CRC in comparison with corresponding normal tissue. *SLC26A3 *mRNA was on average 12.4 times underexpressed (p < 0.01) in CRC than normal surrounding mucosa. Because abnormalities of chromosome 7 have been associated with colorectal carcinoma, we also hypothesised that loss of alleles might lead to underexpression of *SLC26A3*. However, no loss of *SLC26A3 *was detected using the real-time PCR methodology, showing that some other mechanism must be involved in downregulation of the *SLC26A3 *gene.

Decorin is a member of the small leucine-rich proteoglycan gene family. Implication of decorin in the regulation of cell cycle has been suggested by numerous studies. Overexpression of decorin in Chinese hamster ovary cells inhibits cell proliferation [26]. Santra et al. showed that de novo expression of decorin leads to an arrest of transformed cells in the G1 phase of the cell cycle. Moreover, they were able to restore growth by blocking decorin transcription via aDcns [27]. The tumour suppressor activity of decorin is mediated through various pathways. Seidler and co. showed that decorin causes a reduction of activity and decline in total EGFR in tumour cells. Moreover, the same group showed that decorin also activates caspase-3, the key enzyme in apoptosis [28]. Decorin causes the induction of p21, which in turn leads to growth suppression [29]. Decorin also has binding sites for TGF-β, through which it apparently modulates the action of this important citokine [30]. Interestingly, although many tumour suppressor effects have been associated with decorin, no precise quantification by real time PCR has been performed. However, decorin protein under-expression in CRC has been clearly established using immunohistochemistry [31]. We are the first to report and characterise decorin expression on the mRNA level using a real-time PCR quantification method. Our results show that the *DCN *gene is 4.6 times (p < 0.01) underexpressed in tumour than normal tissue. However, in two CRC samples we found decorin overexpression in comparison to normal tissue. Because Decorin acts through EGFR, we speculated that we might see a difference in *DCN *expression between CRCs with *KRAS *mutations and CRCs without them. We found that CRCs with activating mutations in the *KRAS *gene showed much larger underexpression of the *DCN *gene in comparison to normal tissue than CRCs without mutations in *KRAS*. In the Fearon and Vogelstein model, activating KRAS mutations are one of the first genomic events leading to the development of CRC. This in turn suggests that underexpression of the *DCN *gene might be early consequence of acquired activating *KRAS *mutations [32]. Moreover, as reported previously, overexpression of decorin also plays a role in TGF-β by inhibiting its synthesis and bioactivity, thus entering another important CRC development pathway as a tumour suppressor gene [30]. The importance of decorin downregulation for the development of CRC is further emphasized by its downregulation despite the absence of activating KRAS mutations. Lastly, we found no deletions of the *DCN *gene using the real-time PCR method, showing that either methylation of promoter or suppression by trans-activating elements must be responsible for *DCN *underexpression in a tumour.

Tropomyosines are important actin binding proteins. The actin cytoskeleton plays an important role in regulation of cell proliferation, apoptosis, cell migration, invasion and anchorage independent growth [33]. Tropomysine 1 is consistently abolished in a number of human cancer cell lines and tissues [34,35]. Moreover, it has been demonstrated that tropomysin 1 (*TPM1*) has tumour suppressor properties [36] and it can revert transformed phenotype [37]. Despite the obvious importance of *TPM1 *in tumour development, data on *TPM1 *gene expression in clinical samples of CRC samples in comparison to corresponding normal tissue do not exist. Our data obtained by real-time PCR show 1.6 times lower expression of *TPM1 *in CRCs, which supports the current view of *TPM1 *as a tumour suppressor. *TPM1 *was underexpressed in 9 out of 16 cases, in 6 samples no major difference between CRC and normal tissue was found and in 1 case we found *TPM1 *overexpression. More interesting was the observation that *TPM1 *was much more underexpressed in CRCs not harbouring *KRAS *mutations in comparison to normal tissue. In CRCs with a *KRAS *mutation, we found almost no difference in expression to normal tissue. This observation suggests the conclusion that downregulation of *TPM1 *is independent of the constitutively activated *KRAS *pathway. This is also in agreement with the observation that epigenetic factors such as methylation of the promoter region cause *TPM1 *downregulation. Moreover, we also report identification of *TPM1 *gene loss in 1 CRC, showing that loss of genetic material could also be responsible for underexpression of the *TPM1 *gene. This is in agreement with the hypothesis that epigenetic and genetic events are responsible for *TPM1 *downregulation, rather than inhibition by trans acting elements.

One of the interesting results from microarrays was underexpression of the *CALM3 *gene, which codes for calmodulin protein. Since expression of calmodulin 3 was variable among different microarray studies (see additional file 1) we validated our result with real-time PCR and confirmed the observed underexpression of *CALM3 *in CRC. However, this result is in contrast with its proposed cell cycle promotion role, which is reflected by the arrest of the mitotic cycle by anti-CaM drugs added to a wide variety of cells [38]. Moreover, addition of monoclonal antibody against CaM inhibited DNA synthesis and progression through G1, and mitosis exit was sensitive to intracellular concentrations of CaM [39]. Calmodulin in higher vertebrates is encoded by three genes, although all three genes code for an identical calmodulin protein consisting of 148 amino acids [40]. This raises the question of whether the other two CALM genes are expressed in a different way. Surprisingly, both *CALM1 *and *CALM2 *showed significant underexpression, as reported by various microarray studies [14-17]. This observation supports the hypothesis proposed by Kahl and Means that cancer cells containing a disruption in the cyclin D/cdk4 7 pRb regulatory pathway, may no longer require Ca2+/CaM to regulate the activation of this pathway because it is already activated or unnecessary for G1 progression in the tumour cells [38]. One other possible explanation of such a result is that the position in the cell rather than the amount of calmodulin is enough to promote cell cycle. The cell could achieve this positioning of calmodulin by targeting CALM mRNAs to a different intracellular compartment [41,42].

## Conclusion

In conclusion, we detected significant under-expression of genes *SLC26A3*, *TPM1*, *DCN *and *CALM3 *in CRC, providing further evidence of their decreased mRNA expression and thus implicating them in the development of this type of cancer. We also described a correlation between *TPM1 *and *DCN *expression and the presence of *KRAS *mutations in CRC. When searching for chromosomal abnormalities, we found deletion of the *TPM1 *gene in one case of CRC, and no deletions of *DCN *and *SLC26A3 *were found. Moreover, we found underexpression of the *TPM1 *gene in cases of CRCs without *KRAS *mutations, showing that *TPM1 *might serve as an alternative path of development of CRC. This downregulation could in some cases be mediated by deletion of the *TPM1 *gene. On the other hand, the correlation of *DCN *underexpression with the presence of *KRAS *mutations, suggests that *DCN *expression is affected by the presence of activating *KRAS *mutations, lowering the amount of the important tumour suppressor protein decorin.

## Competing interests

The authors declare that they have no competing interests.

## Authors' contributions

VM carried out the analysis of *KRAS *mutations, real-time PCR and wrote and prepared the manuscript. VM and GB carried out microarray and statistical analysis and participated in the design of the study. ZŠ and MR did the surgery and obtained samples and informed consents from patients. MV helped with pathological evaluation of tissue samples and histological staging. DG conceived the study, and participated in its design and coordination and helped to draft the manuscript. All authors read and approved the final manuscript.

## Pre-publication history

The pre-publication history for this paper can be accessed here:

http://www.biomedcentral.com/1471-2407/9/282/prepub

## Supplementary Material

Additional file 1 **Table 2 Differentially expressed genes in 16 colorectal cancers in direct comparison to corresponding normal tissue**. The column, Independent validation, contains the result of a search for articles describing particular gene expression on the level of mRNA in CRC, done with methods other than microarray. References 14–18 were used to compare gene expression. Click here for file
