# Supplementary material for: Presence of activating KRAS mutations correlates significantly with expression of tumour suppressor genes DCN and TPM1 in colorectal cancer
Source: BMC Cancer. 2009 Aug 13;9:282. doi: 10.1186/1471-2407-9-282 (PMC2745427; doi:10.1186/1471-2407-9-282)
Supplement: Additional file 1 — Table 2 Differentially expressed genes in 16 colorectal cancers in direct comparison to corresponding normal tissue. The column, Independent validation, contains the result of a search for articles describing particular gene expression on the level of mRNA in CRC, done with methods other than microarray. References 14–18 were used to compare gene expression. [file 1471-2407-9-282-S1.doc]

Table 2 Differentially expressed genes in 16 colorectal cancers in direct comparison to corresponding normal tissue

| **Gene Symbol** | **Gene Name** | **Fold change** | **Down** | **Up** | **No change** | **Independent validation** |
| --- | --- | --- | --- | --- | --- | --- |
| **Under-expression** | | | | | | |
| SLC26A3 | CHLORIDE ANION EXCHANGER | -2,22 | 16,14 |  |  | no |
| *CASP9* | CASPASE9 PRECURSOR | -1,89 | 17,18 |  | 14 | no |
| *VIM* | VIMENTIN | -1,82 | 16,17 |  | 14 | no |
| *AD022* | TRAF AND TNF RECEPTOR-ASSOCIATED PROTEIN | -1,79 | / | / | / | no |
| *LGALS4* | GALECTIN4 | -1,75 | 14,17 |  |  | yes |
| *SRI* | SORCIN | -1,72 | 16,14,17 |  |  | no |
| *DCN* | DECORIN PRECURSOR | -1,72 | 17 |  |  | no |
| *UGP2* | UTPGLUCOSE1PHOSPHATE URIDYLYLTRANSFERASE 2 | -1,69 | 16,14,18 |  |  |  |
| *ITGB4* | INTEGRIN BETA4 PRECURSOR | -1,61 |  | 14 | 16,17,15 | yes |
| *LGALS3* | GALECTIN3 | -1,56 | 16,14,17,15 |  |  | yes |
| *CDH12* | BRAIN CADHERIN PRECURSOR | -1,49 |  | 16,14 |  | yes |
| *TPM1* | TROPOMYOSIN 1 ALPHA CHAIN | -1,47 | 16,14,17,15 |  |  | no |
| *CALM3* | CALMODULIN | -1,43 | 17 | 14 | 16,15 | no |
| *EEF1A1* | ELONGATION FACTOR 1 ALPHA 1 | -1,41 | 17 | 16 | 18,15 | no |
| **Over-expression** | | | | | | |
| TUBB | TUBULIN BETA5 CHAIN | 1,42 | 18 | 16,14,17,15 |  | no |
| *MAP2K7* | MITOGEN ACTIVATED PROTEIN KINASE KINASE 7 | 1,42 |  | 14,18 | 17 | no |
| *PMP22* | PERIPHERAL MYELIN PROTEIN 22 | 1,43 | 16,14,15 |  |  | no |
| *NPC1* | NIEMANNPICK C1 PROTEIN PRECURSOR | 1,46 |  |  | 14 | on |
| *HOXA4* | HOMEOBOX PROTEIN HOXA4 | 1,52 | 14 |  | 16,17 |  |
| *PCDH1* | PROTOCADHERIN 1 PRECURSOR | 1,52 |  |  | 16,14,15 |  |
| *CORT* | CORTISTATIN PRECURSOR | 1,53 | / | / | / | no |
| *CARD10* | CASPASE RECRUITMENT DOMAIN PROTEIN 10 | 1,55 | / | / | / | no |
| *KRT19* | KERATIN, TYPE I CYTOSKELETAL 19 | 1,61 | 16,17 |  | 14 | yes |
| *CRABP2* | RETINOIC ACIDBINDING PROTEIN II | 1,68 |  | 14,17 |  | no |
| *GATA2* | ENDOTHELIAL TRANSCRIPTION FACTOR GATA2 | 1,69 | 14 | 17 | 18 | no |
| *TGFBI* | TRANSFORMING GROWTH FACTOR, BETA INDUCED | 1,72 | 14 | 16,17 |  | yes |
| *RAI1* | RETINOIC ACID INDUCED 1 ISOFORM 2 | 1,77 |  | 17 |  | no |
| *RBL2* | RETINOBLASTOMA LIKE PROTEIN 2 | 1,85 |  | 16 | 17 | no |
| *TTYH1* | TWEETY HOMOLOG 1 | 2,31 |  |  | 18 | no |
| *TNA* | TETRANECTIN PRECURSOR (TN) | 2,50 | 16 | 14,17 |  | no |

The column, Independent validation, contains the result of a search for articles describing particular gene expression on the level of mRNA in CRC, done with methods other than microarray. References 14-18 were used to compare gene expression.
